# Texas

**Published:** 1897-03

**Authors:** 


*Texas.* About 3500 sheep admitted at El Paso from Mexico were ordered back, on account of the presence of scab, by an inspector of the Bureau of Animal Industry. Owing to the duty involved some indisposition was shown to returning them, and their slaughter was ordered. Lawsuits are likely to follow. About 3 per cent. were said to be affected.

